# Current Therapeutic Opportunities for Estrogen Receptor Mutant Breast Cancer

**DOI:** 10.3390/biomedicines12122700

**Published:** 2024-11-26

**Authors:** Murugesan Palaniappan

**Affiliations:** 1 Department of Pathology & Immunology, Baylor College of Medicine, Houston, TX 77030, USA; palaniap@bcm.edu; 2 Center for Drug Discovery, Baylor College of Medicine, Houston, TX 77030, USA

**Keywords:** breast cancer, estrogen receptor mutants, endocrine-resistant, Y537S, D538G

## Abstract

Estrogen receptor α (ERα) drives two out of three breast cancers and therefore ERα is a major therapeutic target for ER-positive breast cancer patients. Drugs that inhibit ERα activity or block estrogen synthesis in the body are currently being used in the clinic to treat ER-positive breast cancer and have been quite successful in controlling breast cancer progression for the majority of patients. However, ER-positive breast cancer often becomes resistant to these endocrine therapies, leading to endocrine-resistant metastatic breast cancer, a very aggressive cancer that leads to death. Recent large-scale genomic studies have revealed a series of activating somatic mutations in the ERα gene (ESR1) in endocrine-resistant metastatic breast cancer patients. Of these, Y537S and D538G mutations are found at a much higher rate in patients with metastatic breast cancer. Remarkably, these mutations produce an ERα with much higher transcriptional activity than wild type in the absence of estradiol, and traditional endocrine therapy has poor efficacy against ER mutants. Therefore, the development of new drugs that target ER mutants is an unmet clinical need for endocrine-resistant metastatic breast cancer. This review summarizes the recent preclinical and clinical trials targeting estrogen receptor mutant breast cancer.

## 1. Introduction

Breast cancer is the most frequently diagnosed cancer in women and the second leading cause of cancer death in women. There are more than 2.2 million new cases of breast cancer each year worldwide. The future burden of breast cancer is expected to increase to over 3 million new cases and 1 million deaths in 2040 [1]. Breast cancer is highly heterogeneous with distinct molecular features. It can be categorized as either positive or negative for the immunohistochemical expression of three hormone receptors: the estrogen receptor (ER), the progesterone receptor (PR), and the human epidermal growth factor receptor 2 (HER2); cancer may be positive or negative for any of these, and those that are negative for all three are termed triple-negative breast cancer (TNBC). Four main molecular subtypes are also used in the research settings of breast cancer: Luminal A, Luminal B, HER2-enriched, and triple-negative/basal-like. Luminal A breast cancer is the most frequently diagnosed molecular subtype and makes up about 50 to 60% of all breast cancer cases [2]. Luminal A breast cancers express estrogen receptors and progesterone receptors and are therefore commonly called hormone receptor-positive (HR+) breast cancer. Luminal B breast cancers account for about 15 to 20% of all breast cancer cases. Luminal B breast cancer is also HR+ and can be either HER2-positive (HER2+) or HER2-negative (HER2−). Luminal B breast cancer also expresses a high level of Antigen Kiel 67 (Ki-67). Ki67 is a cell proliferative marker and high expression is associated with lower survival rates [3]. HER2-enriched breast cancers make up about 10 to 15% of all breast cancer cases and may be HR+ or HR−. TNBC represents 10% of all breast cancer cases.

In 1962, Jenson and Jacobsen were the first to describe that the biological effects of estrogen are arbitrated through a receptor that was subsequently identified by Toft and Gorski [4]. ERα was cloned in 1986 [5,6]. Subsequently, ERβ was cloned from rat prostate in 1996 [7]. ERα and ERβ are members of the nuclear hormone receptor superfamily expressed from separate genes that act as transcription factors that regulate gene networks in various biological processes including cell growth and proliferation in target tissues [8]. ERα and ERβ have different tissue expression patterns in both humans and rodents [9]. Of these two receptors, ERα participates in a major role in mammary gland development, function, and tumorigenesis [10,11]. ERα is expressed in a subset of luminal epithelial cells of the mammary gland and regulates mammary epithelial cell proliferation and ductal morphogenesis in the course of postnatal mammary gland development [12]. Both receptors are primarily regulated by estradiol, an 18-carbon steroid with an aromatic A-ring that is predominantly synthesized in the ovary.

**Figure 1:**
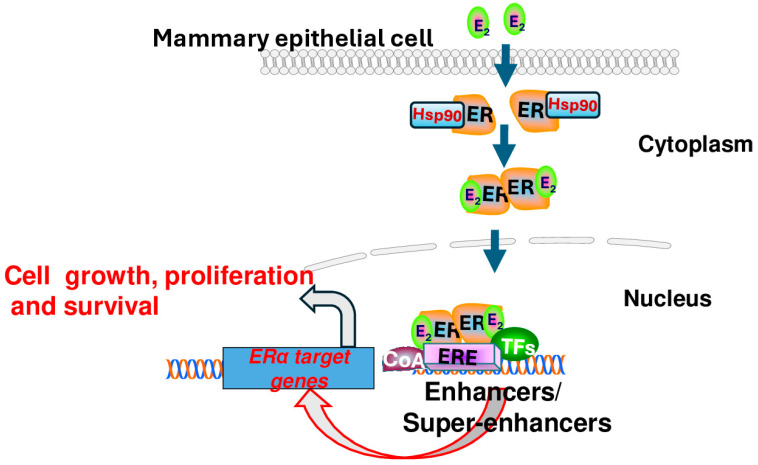
Estradiol-mediated activation of estrogen receptor signaling in mammary epithelial cells. Estradiol (E2) activates estrogen receptor α by displacing heat shock protein 90 (Hsp90), leading to dimerization, translocation from cytosol to nucleus, and binding to estrogen response elements (ERE) in the genome. These estrogen receptor (ER) dimers recruit other transcription factors (TFs) and coactivators (CoAs) to form transcriptionally active complexes at gene enhancers, leading to increased ER-dependent transcription, which drives cell growth, proliferation, and survival.

Estradiol exerts its physiological actions by binding to ERα in the cytoplasm of the breast epithelial cell. Upon binding, heat shock protein 90 (Hsp90) is displaced, and ERα dimerizes and translocates from the cytosol to the nucleus, where ERα dimers can bind to specific estrogen response element (ERE) sequences in the genome and recruit coactivators (CoAs) to form transcriptionally active complexes that directly drive the expression of genes that promote cell growth, proliferation, and survival (Figure 1). Estrogen receptor complexes can also regulate gene transcription via tethering to other transcription factors such as activating protein 1 (AP-1) without binding to ERE sequences (non-genomic effects). AP-1 is involved in a wide range of cellular processes such as cell growth, proliferation, and survival [13,14]. Tertiary ER-regulated genes lack ERE sequences, and the ER-mediated non-genomic signaling that occurs through protein–protein interactions with other transcription factors at their respective response elements affects a broad range of estrogen-regulated genes in breast epithelial cells [15].

## 2. ERα Domain Structure

**Figure 2:**
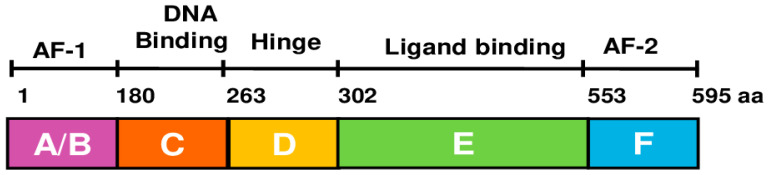
Schematic representation of estrogen receptor α domain structure.

ERα is encoded by the gene ESR1 on chromosome 6 (6q25.1) [16]. ERα comprises 595 amino acids and six structural domains, namely A to F (Figure 2) [17]. The amino-terminal A/B domain contains the transcriptional activation function (AF)-1, which interacts with coregulators and enhances gene transcription in the cell. The A/B domain contains serine phosphorylation sites at positions 104, 106, 118, and 167 that are mainly modified in response to insulin-like growth factor 1, epidermal growth factor, and tumor growth factor α. Phosphorylation of Ser 118 can lead to ligand-independent activation through conformational changes in the A/B domain [18,19]. The C domain, also called the DNA binding domain (DBD), contains two zinc-finger structures that interact with DNA and influence ER dimer formation. The C domain is highly conserved and responsible for binding to specific estrogen response element (ERE) regions of estrogen-regulated genes [20]. The D domain, also known as the hinge region, contains phosphorylation, acetylation, and sumoylation sites that regulate transcriptional activity [21,22]. Lysines 302 and 303 in the hinge region protect ERα from basal degradation [23]. The E domain, also called the ligand-binding domain (LBD), contains 11 alpha-helices (H1, H3–12) in a globular assembly with a deep pocket as reported in 1997 with the first crystal structure of the ERα LBD with the natural ligand 17β estradiol [24]. In response to estradiol, helices 3, 4, 5, and 12 of the LBD form a cleft where coactivators containing the leucine-X-X-leucine-leucine (LxxLL) motif can bind to trigger gene transcription [25]. When partial agonists and antagonists bind, the LBD helix 12 shifts, altering the cleft and blocking interaction with the coactivator LxxLL motif [26]. Helix 12 repositioning in response to binding of fulvestrant leads to the degradation of ERα [27,28,29]. Fulvestrant is a selective estrogen receptor degrader (SERD) and is used to treat advanced ER+ breast cancer. The 45 residue F domain, also known as the carboxyl-terminal domain, is unique to ERs among nuclear receptors. The ERα F domain has 45 amino acids. The F-domain is required for the partial agonist activity of tamoxifen and the effectiveness of the E2-induced transcriptional activity function [30,31,32,33].

## 3. ERα in Human Breast Cancer

In normal breast tissue, ERα is expressed in only around 10% of the epithelial cells. However, in the case of breast tumors, ERα is highly expressed (50 to 80%) [34]. ERα is a major oncogenic driver of breast cancer initiation and progression. Approximately 70% of breast cancers express ERα; since estrogen-mediated ERα signaling plays a central role in the progression of these cancers, ERα is a prime therapeutic target for ER-positive breast cancer patients. In response to estradiol, ERα regulates several thousand genes in breast cancer cells that influence cancer cell growth and proliferation [35,36]. Genome-wide ERα chromatin immunoprecipitation followed by high-throughput sequencing (ChIP-seq) studies showed only a small fraction of ERα binding sites are distributed in the proximal promoter region: most ERα binding sites are located in the distal areas of ER target genes [36,37,38,39]. Brown and colleagues showed that ERα binding regions are highly enriched for the FOXA1 binding motif, and subsequently, FOXA1 was identified as an important pioneer factor for ER–chromatin interactions in ER-positive breast cancer cells [40] and as necessary for E2-mediated gene expression [39]. Carroll and colleagues mapped the ERα binding sites in primary breast samples and showed that different ERα binding profiles are linked with different clinical outcomes of breast cancer [41].

### 3.1. Endocrine Therapy in ER+ Breast Cancer

Endocrine therapy has been one of the most effective treatments for both early and advanced-stage ER+ breast cancer [42,43,44]. Endocrine drugs inhibit ERα activity or block estrogen synthesis in the body. Currently, three types of drugs are used in the clinic to treat ER+ breast cancer: selective estrogen receptor modulators, aromatase inhibitors, and selective estrogen receptor degraders.

**Figure 3:**
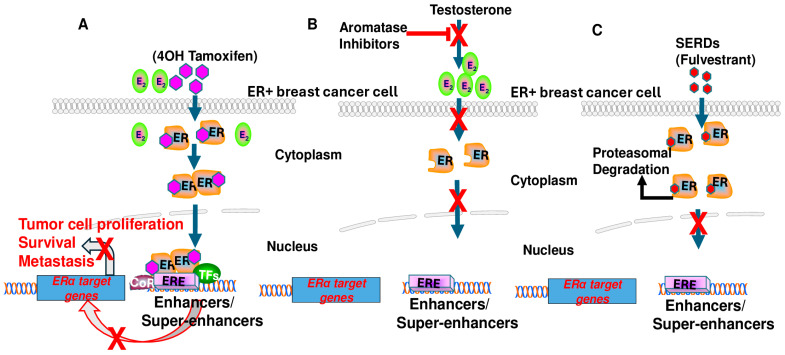
Current clinical approaches to inhibit estrogen receptor α signaling in ER+ breast cancer cells. (**A**) 4OH tamoxifen competitively blocks the binding of estradiol to estrogen receptor α. The 4OH tamoxifen and estrogen receptor α complex translocate from the cytosol to the nucleus, bind to estrogen response elements (EREs) in the genome, and recruit corepressors (CoR) to inhibit E2-induced transcription in ER+ breast cancer cells. (**B**) Aromatase inhibitors inhibit estradiol production by impeding the aromatization of testosterone to estradiol. The loss of estradiol renders estrogen receptor α inactive during treatment with aromatase inhibitors. (**C**) Selective estrogen receptor degraders (SERDs) such as fulvestrant block estrogen signaling by inducing conformations that result in the selective degradation of estrogen receptor α via proteasomal pathways.

Selective estrogen receptor modulators (SERMs) can act as estrogen agonists or antagonists depending on the target tissues. For example, tamoxifen is a classical SERM used widely to treat pre- and postmenopausal women with ER+ breast cancer; it acts as an antagonist in breast tissue, whereas it acts as an agonist in endometrium and bone [45,46]. Tamoxifen was developed in the late 1960s, its first clinical use was reported in 1971 [47], and it was approved by the Food and Drug Administration (FDA) in 1977. Tamoxifen is a non-steroidal drug and has been extensively used to treat ER+ breast cancer. The tamoxifen metabolite 4-hydroxytamoxifen (4-OHT) competes with estradiol (E2) at the ligand-binding site of ER, and 4-OHT-ER complexes that enter the nucleus bind to ERE regions and recruit corepressors, which leads to blocking a subset of E2-inducible genes and inhibits the growth of ER-containing breast cancer cells (Figure 3A). 4-OHT binding to the ERα LBD induces a different conformation than E2 binding, preventing the recruitment of coactivators [48].

Aromatase inhibitors target cytochrome P450 19A1 (aromatase), an enzyme involved in the bio-synthetic conversion of androgens into estrogens. Aromatase inhibitors block estrogen production in the body, which may stop the growth of ER-containing cancer cells that need estrogen to grow. In the presence of aromatase inhibitors, ERs are inactive in the cell (Figure 3B). Three aromatase inhibitors (letrozole, anastrozole, and exemestane) are currently approved by the FDA and used in the clinic to treat early locally advanced and metastatic ER+ breast cancer [49]. They may also be used to help prevent breast cancer in patients who are at a high risk of developing it. Letrozole is a non-steroidal drug that targets the aromatase active site [50].

Selective estrogen receptor degraders (SERDs) fulvestrant and elascestrant are the only drugs approved by the FDA to treat advanced or metastatic ER+ breast cancer. SERDs act as ERα antagonists and also cause ERα degradation in cells. Fulvestrant is a steroid drug administered intramuscularly once monthly and was approved by the FDA in 2002. It is a 7*α*-alkylsulphinyl analogue of 17*β*-estradiol and acts as a pure antiestrogen that inhibits ERα dimerization and nuclear translocation and leads to accelerated ERα degradation through the proteasomal degradation pathway (Figure 3C) [51,52]. These effects completely block Erα-induced transcriptional activity in breast cancer cells, which in turn inhibits tumor progression, invasion, angiogenesis, and metastasis [51,53,54,55]. Elacestrant, a non-steroidal small-molecule drug, was approved by the FDA in 2023 for the treatment of ER-positive, HER2-negative, ESR1-mutated advanced or metastatic breast cancer [56]. In an ER+ breast cancer cell line model, elacestrant induces ERα degradation through the proteasomal pathway, blocking the activity of E2-regulated genes associated with breast cancer cell growth and proliferation [57]. The antitumor effects of elacestrant have been shown in preclinical studies and clinical trials using several prognostic and predictive markers [57,58,59]. Elacestrant binds preferentially to ERα over ERβ with half-maximal inhibitory concentrations of 48 vs. 870 nmol/L [60]. It has also demonstrated antitumor activity in ER-positive patient-derived xenograft (PDX) tumor models with ESR1 mutations and with models for cyclin-dependent kinase 4/6 (CDK4/6) inhibitor resistance [57,61].

### 3.2. Endocrine-Resistant Breast Cancer

Antiestrogen therapy has been one of the most successful therapeutic approaches in ER+ breast cancer. Most ER+ breast cancer patients initially responded well to endocrine therapy that attenuates ERα signaling, either by blocking the production of estrogens via aromatase inhibitors or antagonizing the activity of estrogens by competitive binding of ER antagonists such as tamoxifen and fulvestrant, a SERD [62,63,64,65,66,67]. Although estrogen-disrupting therapies are often effective both in the adjuvant and metastatic setting, patients relapse after prolonged endocrine therapy, which remains a major clinical problem [68,69]. Several mechanisms of endocrine resistance have been identified, including loss of ERα expression, altered activity of ERα coactivators, and crosstalk with growth factor receptors such as HER2 and IGF1R [28,70,71,72,73,74,75,76,77]. Studies have shown that the growth factor-driven mitogenic pathway can drive ER-mediated gene transcription in the absence of estradiol. Furthermore, genomic alterations in the ESR1 gene itself represent a common mechanism of endocrine resistance. Indeed, recent large-scale genomic studies have revealed a series of activating somatic mutations in the ER gene (ESR1) in endocrine-resistant metastatic breast cancer (MBC) patients [78,79,80,81,82,83,84,85,86]. This review highlights the recent preclinical and clinical trials of drugs targeting estrogen receptor mutant breast cancer.

### 3.3. ESR1 Mutation

In 1997, Fuqua and colleagues were the first to identify an ESR1 mutation (Y537N) in the ligand-binding domain of ERα in metastatic ER+ breast cancer patient samples. Nevertheless, the significant role of ESR1 mutations in endocrine resistance was not entirely recognized until 2013, when two independent studies confirmed that somatic ESR1 mutations are relatively common (10–50%) in endocrine therapy-resistant metastatic ER+ breast cancer patients [78,80]. These somatic mutations are found predominantly in the ERα ligand-binding domain (LBD) and the ERα mutations Y537S or D538G, that are present in up to 40% of endocrine therapy-resistant metastatic breast cancer patients and are a major mechanism of acquired resistance to hormonal therapies [82,87,88,89,90,91]. These mutations produce an ER with high transcriptional activity even in the absence of estradiol and with a weaker affinity for anti-estrogens such as tamoxifen and fulvestrant that allows cells to resist these drugs [82,87,88,89,92,93,94,95]. Through conformational biases, mutant ERα proteins induce transcriptional gene activities that are linked with aggressive disease [96,97]. For instance, structural studies indicate that the Y537S mutation stabilizes the agonist conformation in the absence of ligand by forming a hydrogen bond between S537 and D351 that cannot form in WT ERα [98,99]. Y537S and D538G mutations promote constitutive binding of steroid receptor coactivator 3 (SRC3) to these ERα mutant receptors in the absence of ligand, with Y537S recruiting SRC3 coactivator with higher affinity than D538G. Ligand-binding assays reveal that these mutants have weaker affinity than wild type (WT) for estradiol and the antiestrogens tamoxifen and fulvestrant.

Studies in breast cancer cell lines demonstrated that Y537S and D538G mutations produce high levels of ERα transcription activities even in the absence of estradiol, leading to drug resistance [78,99]. Further, these mutant ERαs produce neomorphic transcriptional activities that lead to the expression of genes associated with aggressive disease in genetically engineered breast cancer models [100]. Y537S and D538G mutant breast cancer cells show increased cell proliferation over WT in response to insulin-like growth factor [101]. A genome-wide ERα binding study showed that cells expressing WT protein occupy a small number of DNA binding sites in the absence of estradiol compared to the ERα mutants, leading to distinct alterations in the ER binding pattern. Motif analysis revealed that the mutant-specific ER binding sites are highly enriched in ERE motifs, whereas the WT sites are enriched in FOXA1 motifs, indicating that FOXA1 is essential for WT-specific ER DNA binding but not for mutant ER DNA binding. Transcriptional changes driven by mutant-specific ER DNA binding substantially promote a metastatic phenotype in mice [96], and these mutations are prognostic of poor outcomes in patients with metastatic disease [102].

### 3.4. Therapeutic Strategies for ESR1 Mutant Breast Cancer

Endocrine therapy plays a central role in treating early and metastatic ER+ breast cancer. The management of ER+ metastatic breast cancer combines endocrine therapy with CDK4/6 inhibitors as the standard-of-care (SOC) treatment. Clinical studies showed that ESR1 mutations that promote ligand-independent activation (e.g., Y537S, D538G) occur predominantly in metastatic or advanced breast cancer patients who were previously treated with estrogen deprivation therapy, especially aromatase inhibitors. Therefore, estrogen deprivation therapy may not be effective in ESR1 mutant breast cancer. Indeed, clinical data suggest reduced efficacy of aromatase inhibitors compared with fulvestrant in patients who have ESR1 mutations in the tumor or circulating tumor DNA (ctDNA) [103]. As cancer cells die, ctDNA is released into plasma in small quantities and provides the opportunity to profile tumors for somatic mutations [104,105]. Blood-based ctDNA is preferred owing to greater sensitivity [105]. The advanced digital droplet PCR (ddPCR) method is a more sensitive method to identify ESR1 mutations in cell-free DNA separated from the plasma [106]. The PALOMA-3 multicenter double-blind, randomized phase 3 trial showed that fulvestrant in combination with palbociclib (CDK4/6 inhibitor) was linked with significant improvement in progression-free survival compared with fulvestrant plus placebo, regardless of the endocrine resistance, hormone receptor expression level, and PIK3CA mutational status. This combination therapy is used as a therapeutic option for patients with recurrent hormone receptor-positive, HER2− metastatic breast cancer that has progressed on previous endocrine therapy [44,107].

More recently, elacestrant has been approved by the FDA for the treatment of postmenopausal women with ER+, HER2− and ESR1 mutant metastatic or advanced breast cancer with disease progression following more than one line of endocrine therapy [108]. Elacestrant is the first orally available small-molecule drug in the SERD class. A multinational phase 3 randomized study (ClinicalTrials.gov identifier: NCT03778931, EMERALD) showed that monotherapy with elacestrant dramatically decreases the risk of cancer progression compared with SOC endocrine therapy in patients with ER+ and HER2− metastatic breast cancer (with or without ESR1 mutations) who had progression after first- or second-line treatment with the combination of endocrine therapy and a CDK4/6 inhibitor [58]. The most common side effects to elacestrant were dyslipidemia, musculoskeletal pain, and nausea; elevated levels of triglycerides, AST, ALT, and creatinine; and reduced levels of hemoglobin and sodium. Other adverse effects such as decreased appetite, diarrhea, headache, abdominal pain, constipation, hot flush, and dyspepsia were also observed in response to elacestrant treatment [109,110].

Preclinical mouse models of breast cancer studies (xenograft and PDX) showed that elacestrant prevents E2-induced tumor growth in ESR1 WT and mutants [57,61]. Furthermore, the anti-tumor activity of elacestrant translates into various PDX models representing intrinsic and acquired CDK4/6 inhibitor resistance [61]. Another study evaluated the efficacy of elacestrant alone or in combination with either palbociclib (CDK4/6 inhibitor) or everolimus (mTOR inhibitor) in a xenograft mouse model. Elacestrant alone attained a significant reduction in tumor growth, but combination treatment with palbociclib or everolimus produced even greater inhibition of tumor growth [54]. Furthermore, in two different PDX models harboring ESR1 mutations, combination treatment with elacestrant and palbociclib effectively stopped tumor growth [57,61].

## 4. SERDs in Clinical Trials

In addition to elacestrant, there are several promising small molecules in various stages of clinical development (Table 1). This review focuses on oral SERDs in phase 3 trials.

### 4.1. Giredestrant (GDC-9545)

Giredestrant is an investigational nonsteroidal oral SERD that binds to the ligand-binding domain and triggers intranuclear ER immobilization before degradation [111]. Giredestrant retains efficacy with ER mutants Y537S and D538G. GDC-9545 induces rapid ERα degradation and anti-proliferation across an ER+ breast cancer cell line panel [112]. In a preclinical model, at low doses, GDC-9545 promotes tumor regressions either as a single agent or combined with a CDK4/6 inhibitor in an ESR1 Y537S mutant PDX and in a wild-type ERα breast tumor model [112]. In the phase Ia/b GO39932 study (ClinicalTrials.gov identifier: NCT03332797), the efficacy and tolerability of giredestrant were studied in ER+ and HER2− locally advanced/metastatic breast cancer patients who previously received endocrine therapy [111]. The primary analysis demonstrated that giredestrant is well tolerated and potentially clinically active as a single agent and in combination with palbociclib for the treatment of patients who have disease progression on prior endocrine therapy, including patients with ESR1 mutations. Hepatotoxicity was reported with giredestrant treatment. The phase II acelERA study (ClinicalTrials.gov identifier: NCT04576455) compares the efficacy and safety of giredestrant with physician’s choice of endocrine monotherapy for ER+, HER2– breast cancer in the second or third line [113]. This study showed that giredestrant treatment improved progression-free survival (PFS) but was not statistically significant with ESR1 mutant tumors. Furthermore, giredestrant trended to a favorable benefit, including in patients with ESR1 mutations. Giredestrant is currently being investigated in phase III trials in ER+, HER2– breast cancer. This randomized, open-label multicenter study will evaluate the efficacy and safety of adjuvant giredestrant compared with endocrine therapy of physician’s choice in participants with medium- and high-risk Stage I-III ER+ and HER2− breast cancer (ClinicalTrials.gov identifier: NCT04961996). Another study will evaluate the efficacy and safety of giredestrant compared with fulvestrant, both in combination with the physician’s choice of a CDK4/6 inhibitor (ClinicalTrials.gov identifier: NCT06065748) in patients with ER+, HER2− advanced breast cancer who have developed resistance to adjuvant endocrine therapy. The results of these ongoing phase 3 trials are not yet available.

### 4.2. Imlunestrant (LY3484356)

Imlunestrant is an investigational orally administered SERD that was developed by Eli Lilly [114]. LY3484356 is a pure ERα antagonist with a highly potent and efficient degrader against wild-type and mutant ER. LY3484356 is also a potent inhibitor of ERα-mediated transcription in vitro and in vivo [115]. It inhibits cell proliferation in wild-type ERα and ESR1 mutant breast cancer cell lines. LY3484356 has maintained persistent target inhibition up to 96 h after the last dose in ESR1 wild-type and ESR1 Y537S mutant xenograft tumors [115]. LY3484356 exhibited substantial tumor growth inhibition and tumor regressions in wild-type ESR1 breast cancer xenograft, as well as ESR1 mutant breast cancer PDX models [115]. Furthermore, LY3484356 has displayed additivity in combination with CDK4/6 inhibitors, mTOR inhibitors, and PIK3CA inhibitors in blocking cell proliferation, as well as tumor growth inhibition in xenograft and PDX models of breast cancer. Phase 1a/1b EMBER (ClinicalTrials.gov identifier: NCT04188548) is a comprehensive, open-label, dose-escalation (phase 1a) trial of imlunestrant followed by several dose-expansion cohorts (phase 1b) examining imlunestrant as monotherapy and in combination with abemaciclib with or without aromatase inhibitors everolimus or alpelisib in ER+ advanced breast cancer and endometrial endometrioid cancer. The overall results demonstrate that imlunestrant has an adaptable safety profile with antitumor activity in ER+/HER2− advanced breast cancer, including in patients with baseline ESR1 mutations and fulvestrant- and/or CDK4/6 inhibitor-refractory disease [114]. Based on these positive results, imlunestran entered into the phase 3 EMBER-3 trial (ClinicalTrials.gov identifier: NCT04975308); this study is currently evaluating imlunestrant compared to standard hormone therapy, as well as imlunestrant plus abemaciclib, in patients with ER+/HER2− locally advanced or metastatic breast cancer who had previously been treated with endocrine therapy.

### 4.3. Camizestrant (AZD-9833)

Camizestrant is an investigational oral SERD that was developed by AstraZeneca [116,117]. Camizestrant shows strong and selective ER degradation and significant antiproliferation activity in ESR1 wild-type and mutant breast cancer cell lines [118]. Camizestrant has demonstrated anti-cancer activity in fulvestrant-resistant ESR1 WT, Y537S, and D538G PDX models [118]. Camizestrant in combination with CDK4/6 inhibitors or PI3K/AKT/mTOR inhibitors showed enhanced efficacy in CDK4/6-sensitive and -resistant models [118,119]. Phase 1 trial SERENA-1 (ClinicalTrials.gov identifier: NCT03616587) has demonstrated that camizestrant is well tolerated and has improved clinical activity as monotherapy or in combination with CDK4/6 inhibitors in ER+/HER2− advanced breast cancer patients who had received one or more previous lines of endocrine therapy including CDK4/6 inhibitors [116]. In the randomized multicenter phase 2 trial SERENA-2 (ClinicalTrials.gov identifier: NCT04214288), the efficacy and safety profiles of oral camizestrant were compared with fulvestrant; camizestrant substantially improved progression-free survival (PFS) versus fulvestrant in post-menopausal patients with ER+ advanced breast cancer who had previously been treated with endocrine therapy. Notably, camizestrant remained more effective than fluvestrant in patients with ESR1 mutations [120]. SERENA-4 (ClinicalTrials.gov identifier: NCT04711252) is an ongoing phase 3 randomized, double-blind study that is assessing the efficacy and safety of camizestrant plus palbociclib versus anastrozole plus palbociclib as first-line therapy for patients with HR+/HER2− advanced breast cancer who have not established systemic treatment for advanced disease. SERENA-6 (ClinicalTrials.gov identifier: NCT04964934) is another ongoing phase 3 trial that evaluates the efficacy and safety of substituting from an aromatase inhibitor to camizestrant, but continuing the same CDK4/6 inhibitors, upon finding of ESR1 mutations in circulating tumor DNA before progression of the disease on first-line therapy for HR+/HER2− advanced breast cancer [117]. 

### 4.4. Vepdegestrant (ARV-471)

Vepdegestrant is an investigational, orally bioavailable proteolysis-targeting chimera (PROTAC) protein degrader that is designed to target and degrade ERα wild-type and mutant proteins. In preclinical studies, vepdegestrant-induced degradation of WT and mutant ERα (including Y537S and D538G) and antiproliferation in a panel of ER+ breast cancer cell lines were observed [121]. Vepdegestrant exhibited dose-dependent tumor regressions in xenograft and PDX models of breast cancer, including Y537S and palbociclib-resistant Y537S [121]. More importantly, a combination with small-molecule inhibitors of CDK4/6 (palbociclib, abemaciclib, or ribociclib), PI3K (alpelisib or inavolisib), or mTOR (everolimus) produced robust tumor regressions in most cases [121]. Collectively, these preclinical studies suggest that dual pathway targeting by vepdegestrant and CDK4/6 or PI3K/mTOR signaling could result in better therapeutic outcomes for patients with advanced ER+/HER2− breast cancer [121]. Based on the encouraging preclinical data, a first-in-human phase 1/2 study (ClinicalTrials.gov identifier: NCT04072952) of vepdegestrant monotherapy and in combination with palbociclib was conducted in ER+/HER2− breast cancer patients; vepdegestrant demonstrated antitumor activity and was tolerated in daily doses from 30 to 700 mg, with no dose-limiting toxicities [122]. Based on the results of the dose escalation study, vepdegestrant 200 and 500 mg were further assessed in VERITAC, the phase 2 expansion cohort of the phase 1/2 study. Preliminary results showed anti-tumor activity, as well as a well-tolerated safety profile [123]. VERITAC-2 (ClinicalTrials.gov identifier: NCT05654623) is a phase 3 study that compares the efficacy and safety of vepdegestrant with fulvestrant in patients with ER+/HER2− advanced breast cancer after prior combination endocrine therapy and CDK4/6 inhibitor therapy [124].

### 4.5. Palazestrant (OP-1250)

Palazestrant is an investigational orally bioavailable SERD and ER antagonist that was developed by Olema Pharmaceuticals. In breast cancer cell lines, palazestrant inhibits estrogen-induced transcriptional activity and blocks agonist activity on estrogen-induced genes, as well as antiproliferative activity in WT and ESR1 mutant cells [125]. In preclinical studies, palazestrant demonstrated tumor regression in ER+ xenograft models, and CDK4/6 inhibitors enhanced its efficacy [125]. In PDX models bearing the ESR1 Y537S mutant, palazestrant inhibits tumor growth at a 3 mg/kg dose and enhances tumor shrinkage at higher doses or combined with CDK4/6 inhibitors. Palazestrant outperformed elacestrant, a clinically FDA-approved drug for patients with ESR1 mutations [125]. In pharmacokinetic analyses of mouse xenograft studies, palazestrant displays excellent brain penetrance and an encouraging half-life [125]. In an intracranial xenograft study, treatment with 10 mg/kg palazestrant demonstrated tumor shrinkage and survival of all animals over a 100-day dosing interval, significantly surpassing the performance of fulvestrant and tamoxifen [125]. Based on the encouraging preclinical data, palazestrant entered into clinical trials. ClinicalTrials.gov identifier NCT04505826 is a phase 1 dose escalation and dose expansion and phase 2 monotherapy trial to assess the dose-limiting toxicity (DLT), maximum tolerated dose (MTD), and/or recommended phase 2 dose, to analyze the safety and pharmacokinetic profile, and to evaluate the preliminary anti-tumor activity of palazestrant as a single agent in adult with ER+/HER2− metastatic breast cancer or locally advanced breast cancer with and without ESR1 mutation. Palazestrant showed an acceptable safety profile, satisfactory pharmacokinetics and encouraging antitumor efficacy in patients with and without ESR1 mutation at the endorsed phase 2 dose of 120 mg once a day. OPERA-01 (ClinicalTrials.gov identifier: NCT06016738) is a multicenter, randomized, phase 3 clinical trial comparing the efficacy and safety of palazestrant as a single agent to endocrine therapies (fulvestrant, anastrozole, letrozole, or exemestane) in patients with ER+, HER2– metastatic breast cancer that relapsed on 1–2 prior lines of endocrine therapies in combination with CDK4/6 inhibitor. This phase 3 trial started last year and is still recruiting patients.

## 5. Conclusions and Future Directions

Endocrine therapies are effective both in the adjuvant and metastatic setting with ER+ breast cancer patients. However, a major clinical problem is that patients often relapse after prolonged endocrine therapy. One of the major mechanisms that drives endocrine-resistant metastatic breast cancers is constitutively active, somatic point mutations in the ligand-binding domain of ERα. The most common ligand-binding domain point mutations are Y537S and D538G, which promote estrogen-independent ER transcriptional activity and decreased sensitivity to current endocrine drugs tamoxifen and fulvestrant. Recent drug discovery efforts for orally available SERDs have led to the identification of several novel investigational agents undergoing clinical evaluation. So far, oral SERD efforts for breast cancer have led to the authorization of elacestrant, the first oral SERD approved for treating metastatic, hormone-resistant breast cancer, including ESR1 mutants. Tackling issues of endocrine resistance to various antiestrogens and anticancer agents will continue to be a critical challenge, and it seems likely that the development of effective combination therapy will be needed to completely combat resistance mechanisms that occur during treatment with new inhibitors. Indeed, the next generation of oral SERDs is currently being evaluated with CDK4/6 inhibitors (palbociclib, ribociclib, and abemaciclib). As an alternative, or a complement, inhibitors of PI3K, AKT and/or mTOR are being combined with SERDs, since abnormal activation of growth factor signaling cascades have been associated with endocrine therapy resistance.

It will also be important to identify and develop first-generation ESR1-mutant-specific inhibitors because elacestrant and other clinical investigational SERDs exhibit lower activity against Y537S and D538G mutants than against wild-type receptors. Drug discovery efforts that use novel and creative strategies to target these mutants will provide a promising new avenue for the direct pharmacological inhibition of ERα mutant proteins in endocrine therapy-resistant metastatic breast cancer patients.

## Figures and Tables

**Table 1:** Next-generation ER-targeting drugs in clinical trials.

Drug	Endocrine Therapy Class and Study Details	Chemical Structure
AND019	Selective ERα degrader (SERD) NCT05187832 Phase 1, Recruiting (Kind Pharmaceuticals)	NOT Disclosed
G1T48	SERD NCT03455270 Phase 1, Completed (G1 Therapeutics)	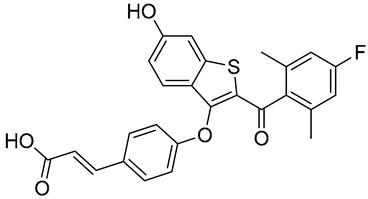
D-0502	SERD NCT03471663 Phase 1, Completed (InventisBio)	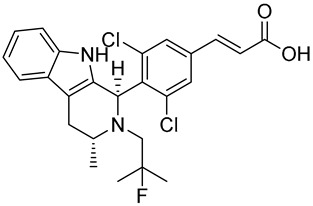
SIM0270	SERD NCT05293964 Phase 1, Recruiting (Jiangsu Simcere Pharmaceutical)	NOT Disclosed
H3B-6545	Selective ERα covalent antagonist (SERCA) NCT03250676 Phase 1, Completed (Eisai)	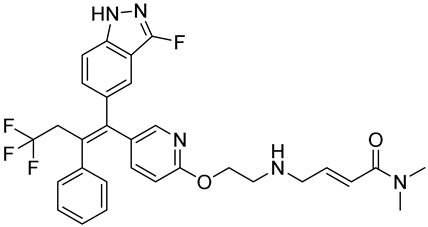
ZN-c5	SERD NCT03560531 Phase1/2, Completed (Zeno Alpha)	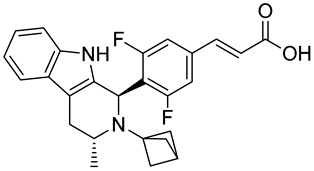
GDC-9545	SERD NCT06065748 Phase 3, Recruiting (Hoffmann-La Roche)	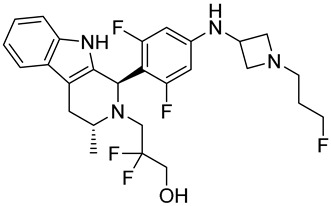
LY3484356	SERD NCT05514054 Phase 3, Recruiting (Eli Lilly and Company)	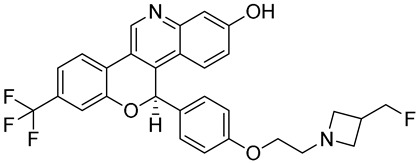
AZD9833	SERD NCT04964934 Phase 3, Active, not recruiting (AstraZeneca)	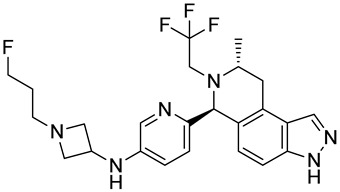
OP-1250	SERD NCT06016738 Phase 3, Recruiting (Olema Pharmaceuticals)	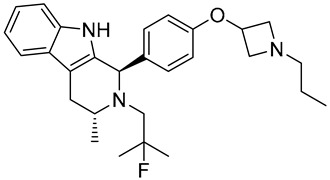
ARV-471 (PF-07850327)	ER PROTAC degrader NCT05654623 Phase 3, Recruiting (Pfizer)	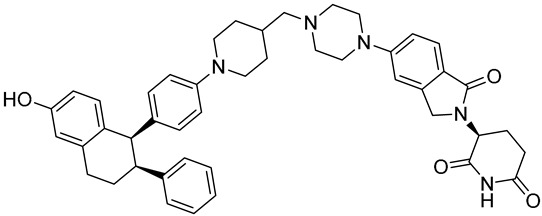

## Data Availability

No new data were created or analyzed in this study. Data sharing is not applicable to this article.
